# The Role of Digital Rectal Examination for Diagnosis of Acute Appendicitis: A Systematic Review and Meta-Analysis

**DOI:** 10.1371/journal.pone.0136996

**Published:** 2015-09-02

**Authors:** Toshihiko Takada, Hiroki Nishiwaki, Yosuke Yamamoto, Yoshinori Noguchi, Shingo Fukuma, Shin Yamazaki, Shunichi Fukuhara

**Affiliations:** 1 Department of Healthcare Epidemiology, School of Public Health in the Graduate School of Medicine, Kyoto University, Kyoto, Japan; 2 Department of General Medicine, Shirakawa Satellite for Teaching And Research (STAR), Fukushima Medical University, Fukushima, Japan; 3 Center for Innovative Research for Communities and Clinical Excellence (CIRC^2^LE), Fukushima Medical University, Fukushima, Japan; 4 Institute for Advancement of Clinical and Translational Science (iACT), Kyoto University Hospital, Kyoto, Japan; 5 Division of General Internal Medicine, Japanese Red Cross Nagoya Daini Hospital, Nagoya, Japan; Centre for Inflammation Research, UNITED KINGDOM

## Abstract

**Background:**

Digital rectal examination (DRE) has been traditionally recommended to evaluate acute appendicitis, although several reports indicate its lack of utility for this diagnosis. No meta-analysis has examined DRE for diagnosis of acute appendicitis.

**Objectives:**

To assess the role of DRE for diagnosis of acute appendicitis.

**Data Sources:**

Cochrane Library, PubMed, and SCOPUS from the earliest available date of indexing through November 23, 2014, with no language restrictions.

**Study Selection:**

Clinical studies assessing DRE as an index test for diagnosis of acute appendicitis.

**Data Extraction and Synthesis:**

Two independent reviewers extracted study data and assessed the quality, using the Quality Assessment of Diagnostic Accuracy Studies 2 tool. Bivariate random-effects models were used for the pooled sensitivity, specificity, positive likelihood ratio, negative likelihood ratio, and diagnostic odds ratio (DOR) as point estimates with 95% confidence intervals (CI).

**Main Outcomes and Measures:**

The main outcome measure was the diagnostic performance of DRE for diagnosis of acute appendicitis.

**Results:**

We identified 19 studies with a total of 7511 patients. The pooled sensitivity and specificity were 0.49 (95% CI 0.42–0.56) and 0.61 (95% CI 0.53–0.67), respectively. The positive and negative likelihood ratios were 1.24 (95% CI 0.97–1.58) and 0.85 (95% CI 0.70–1.02), respectively. The DOR was 1.46 (0.95–2.26).

**Conclusion and Relevance:**

Acute appendicitis cannot be ruled in or out through the result of DRE. Reconsideration is needed for the traditional teaching that rectal examination should be performed routinely in all patients with suspected appendicitis.

## Introduction

Acute appendicitis is one of the most common diagnoses associated with acute abdominal pain, with lifetime prevalence of 7% [1]. Its diagnosis can be challenging because the clinical presentation is often atypical and overlaps with other conditions [2]. Delay of accurate diagnosis could result in rupture of the appendix, which is associated with a worse prognosis [3]. Therefore, a prompt and accurate diagnosis of appendicitis is crucial. Careful history taking and physical examination have an important role in the correct diagnosis [4].

In the physical examination for evaluation of appendicitis, digital rectal examination (DRE) has been considered a necessary investigation [5]. This traditional teaching that DRE should be performed routinely in all patients with abdominal pain has been supported in most surgical textbooks [6,7]. On the other hand, several reports found that DRE rarely provides useful information for diagnosis of acute appendicitis [8,9] and it often induces mild to severe discomfort in patients [10,11].

To date, some review articles investigated the role of DRE for diagnosis of acute appendicitis [8,9,12–15], but none of them used the recommended methods for a meta-analysis of diagnostic studies [16]. Accordingly, the aim of the current study was to perform a meta-analysis of studies that report the diagnostic role of DRE for acute appendicitis to determine the accuracy of this traditional examination.

## Methods

We followed standard guidelines for a systematic review of diagnostic studies [16].

### Data sources and searches

We conducted a literature search of MEDLINE via PubMed, Scopus, and Cochrane Library via Wiley Online Library from the earliest available date of indexing through November 23, 2014. See S1 Text for protocol and searching strategy. The literature search identified potential studies in all languages. We translated the non-English language papers and fully assessed them for potential inclusion in the review as necessary. We checked reference lists of all included studies and relevant systematic review articles for additional references. We contacted authors of additional studies identified that were missed in the original electronic searches.

### Study selection

Studies were included if they met the following conditions: (1) DRE was assessed as an index test (the test under investigation) to evaluate the diagnosis of appendicitis; (2) the reference standard (the criterion standard) of appendicitis was defined as histologically proven acute inflammation in the appendix; (3) exclusion of appendicitis was histologically proven or evaluated in the clinical follow-up with careful observation; and (4) absolute numbers of true-positive, false-positive, false-negative, and true-negative results were reported in the articles, or these data were derivable from the published results.

Two authors (TT and NH) examined the titles and abstracts of references identified by the electronic search strategies described above to check whether the study was likely to be relevant. Studies that were considered potentially relevant in the search were obtained as full articles and independently assessed for inclusion by the same two authors. In the case of discordance, resolution was sought by discussion between the two authors. The discordance in the selection of studies was evaluated by quantifying both the percentage of agreement and Cohen’s Kappa (k). Values of kappa between 0.40 and 0.59 have been considered to reflect fair agreement, between 0.60 and 0.74 to reflect good agreement, and 0.75 or more to reflect excellent agreement [17].

### Data extraction and quality assessment

Two authors used a structured, pilot-tested, Excel data collection form to independently extract the data from the included studies. Extracted data included study characteristics (design, one/two-gate, number of participants, number of excluded participants, index test, reference test, blinding), patient characteristics (setting, age, sex). Two authors also independently assessed methodological quality using the Quality Assessment of Diagnostic Accuracy Studies 2 (QUADAS-2) tool [18]. Any disagreement was resolved by discussion between the two authors. When relevant information regarding design or outcomes was unclear, the original study authors were contacted for clarification.

### Data synthesis and analysis

To evaluate the diagnostic performance, we constructed 2 × 2 tables. Measures of the diagnostic performance, including sensitivity, specificity, positive likelihood ratio (LR+), negative likelihood ratio (LR-), and diagnostic odds ratios (DORs), were reported as point estimates with 95% CI. A DOR can be calculated as the ratio of the odds of positivity in a disease state relative to the odds of positivity in the nondisease state, with higher values indicating better discriminatory test performance. The value of the DOR ranges from zero to infinity, with higher values indicative of better discriminative performance. A value of 1 indicates that the test does not discriminate between people with and without the disease/condition [19]. Publication bias was assessed using the effective sample size funnel plot and associated regression test of asymmetry [20]. We used the bivariate random-effects model for analysis and pooling of the diagnostic performance measures across studies [21]. The bivariate model was used to estimate pairs of logit transformed sensitivity and specificity from studies, incorporating the correlation that might exist between sensitivity and specificity. We used the hierarchical summary receiver operating characteristic (HSROC) curves to estimate the area under the curve [22]. Between-study statistical heterogeneity was assessed using the *I* ^*2*^ test on the basis of the random-effects analysis [23]. To explain heterogeneity, we performed meta-regression to identify potential sources of bias. Pooled estimates were also calculated for subgroups of studies that were defined according to specific study designs. The following variables were selected a priori as potential sources of heterogeneity: publication year, number of participants, study design (prospective vs. retrospective), age of participants (children vs. adults vs. all ages), sex, inclusion criteria (abdominal pain vs. suspected appendicitis/appendectomy), definition of positive DRE (right-sided tenderness vs. the others), reference standard (appendectomy vs. appendectomy/follow-up). The definition of children was adopted in accordance with each article. Two-sided *P* < .05 was considered statistically significant. Statistical analyses were performed with commercial software programs (STATA, version 13.1 SE; StataCorp LP, College Station, Texas).

## Results

### Study selection

**Fig 1:**
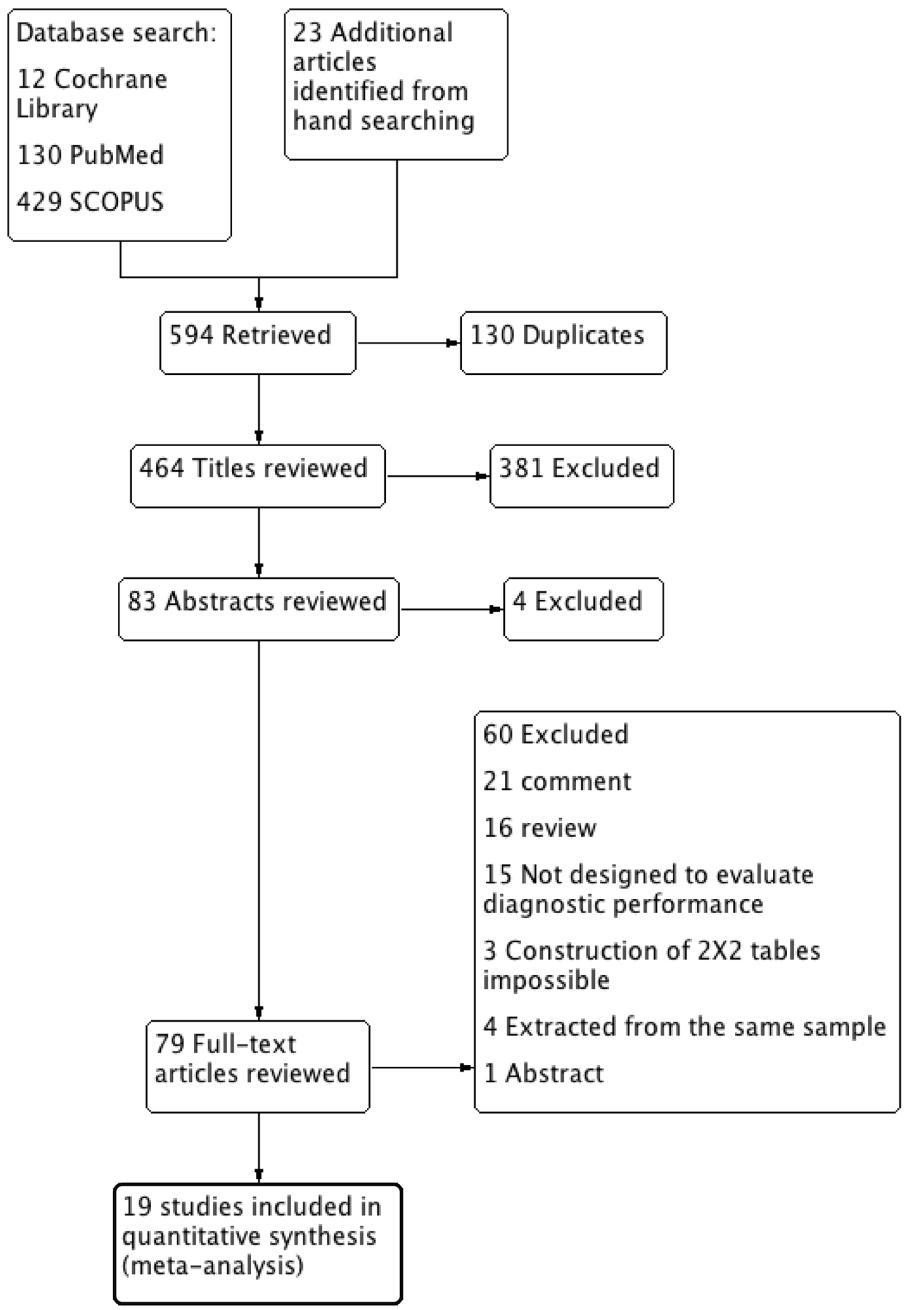
PRISMA flowchart.

We identified 12 articles from the Cochrane Library, 130 from PubMed, 429 from SCOPUS, and 23 from hand-searching. Seventy-nine full-text articles were assessed for eligibility, and 19 studies were included in the final analysis [10,24–45]. The literature search process is shown in Fig 1. The percentage of agreement in the selection of studies was 91.8%, and Cohen’s Kappa (k) was 0.79, which is regarded as excellent agreement [17].

### Characteristics of included studies

**Fig 2:**
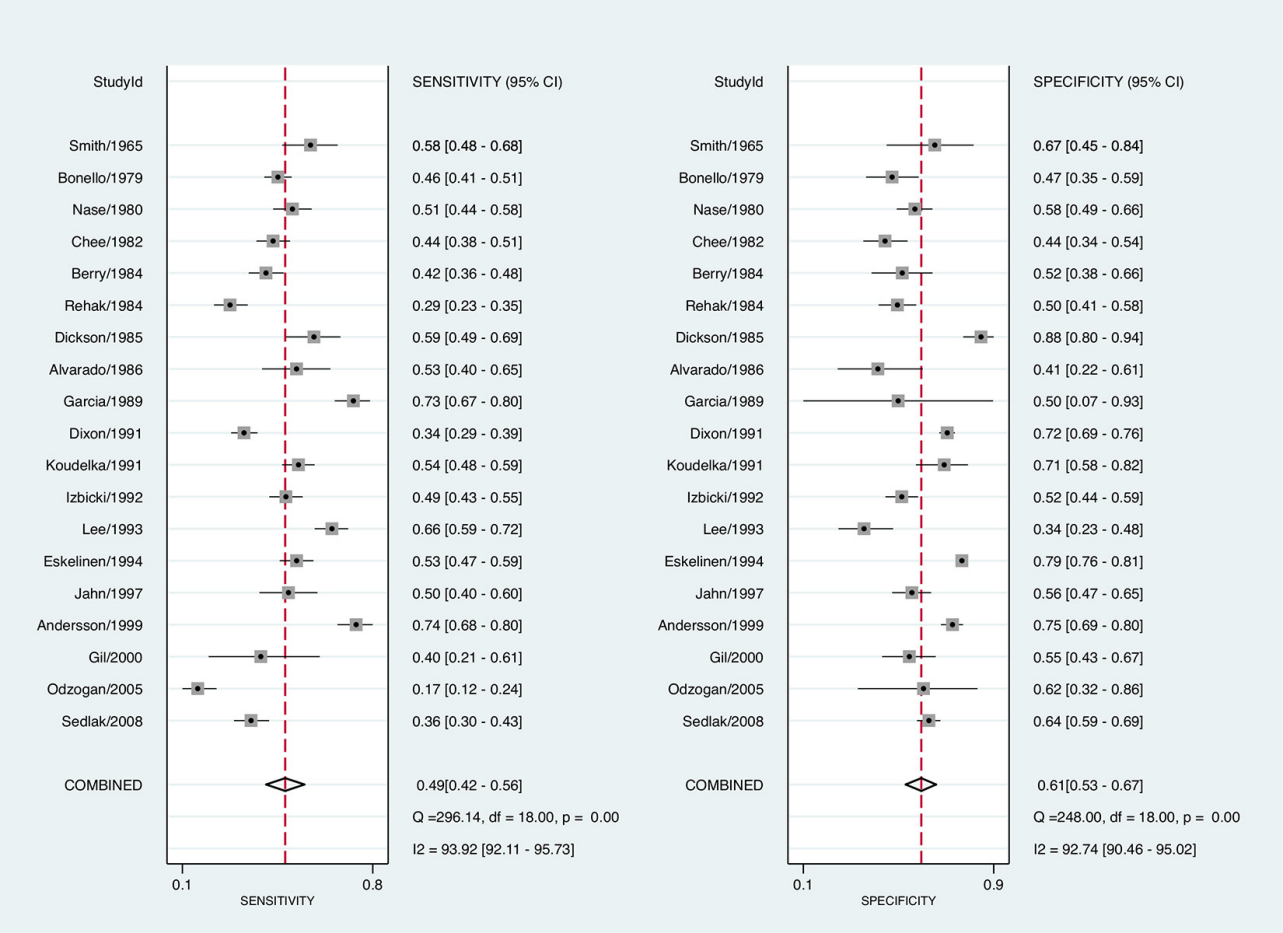
Paired Forest Plots of the Sensitivity and Specificity of DRE for the diagnosis of appendicitis.

A total of 7511 patients were included in the 19 studies. The characteristics and results of studies are summarized in Table 1 and Fig 2. Nine studies were prospective, and 10 were retrospective. Thirteen studies included all age groups, 4 included only adults, and 2 included only children. Inclusion criteria were patients with appendectomy in 8 studies, suspected appendicitis in 8, and abdominal pain in 3 studies. The definition of “positive” DRE was right-sided tenderness for diagnosis of appendicitis [6]. Eight studies defined right-sided tenderness as positive, while 11 studies had no apparent definition, or right-sided tenderness alone was not regarded as positive. With regard to exclusion of appendicitis, 10 studies used a histologic diagnosis, and 9 studies used careful observation for exclusion of appendicitis.

**Table 1:** Clinical features of included studies.

Author	Year	Design	No. of Patients	Age of patients	Patients' characteristics	Definition of positive DRE	Diagnosis of appendicitis	Exclusion of appendicitis
Smith[22]	1965	pro	124	all	appendectomy	tds	histology	histology
Bonello[23]	1979	retro	495	all	appendectomy	right tds, no evidence of other disease	histology	histology
Nase[24]	1980	retro	359	all	suspected appendicitis	right tds	histology	histology/follow-up
Chee[25]	1982	pro	370	>12	appendectomy	right tds, no evidence of other disease	hitology	histology
Berry[26]	1984	retro	307	all	appendectomy	tds	histology	histology
Rehak[27]	1984	retro	364	all	appendectomy	right tds	histology	histology
Dickson[10]	1985	pro	201	<14	suspected appendicitis	anterior or right tds, sweling or a mass	histology	histology/follow-up
Alvarado[28]	1986	retro	305	all	suspected appendicitis	right tds	histology	histology/follow-up
Garcia[29]	1989	pro	200	all	appendectomy	right tds	histology	histology
Dixon[30]	1991	retro	1204	all	right lower abdominal pain	right tds	histology	histology/follow-up
Koudelka[31]	1991	retro	402	< = 15	suspected appendicitis	pain in Douglas	histology	histology
Izbicki[32]	1992	retro	536	all	appendectomy	anterior/posterior/right/left/mass	histology	histology
Lee[33]	1993	retro	555	all	appendectomy	tds	histology	histology
Eskelinen[34–38]	1994	pro	222	all	acute abdominal pain<7 days	tds	histology	histology/follow-up
Jahn[39]	1997	pro	212	all	suspected appendicitis	tds	histology	histology/follow-up
Andersson[40]	1999	pro	502	> = 10	suspected appendicitis	right tds	histology	histology/follow-up
Ödzogan[41]	2005	pro	170	> = 16	suspected appendicitis	tds	histology	histology
Sedlak[42]	2008	retro	659	>16	right lower abdominal pain	right, left, cranial side or generalized	histology	histology/follow-up

^a^ tenderness

### Quality of included studies

S1 Fig and S2 Fig present an overview of the quality of included studies evaluated by QUADAS-2. In terms of patient selection, 3 studies had inappropriate exclusions. The index test was judged as unclear in 6 studies that did not describe the definition of “positive” DRE. In terms of flow and timing, 9 studies that excluded appendicitis by histology or careful observation were regarded as having a high risk of bias. Most studies did not describe whether the result of histopathology and final diagnosis in follow-up were interpreted without knowledge of results of DRE. In S3 Fig, the funnel plots and regression test indicate no significant publication bias (*P* = 0.47), although it seemed that more studies reported a low DOR.

### Overall diagnostic accuracy

**Fig 3:**
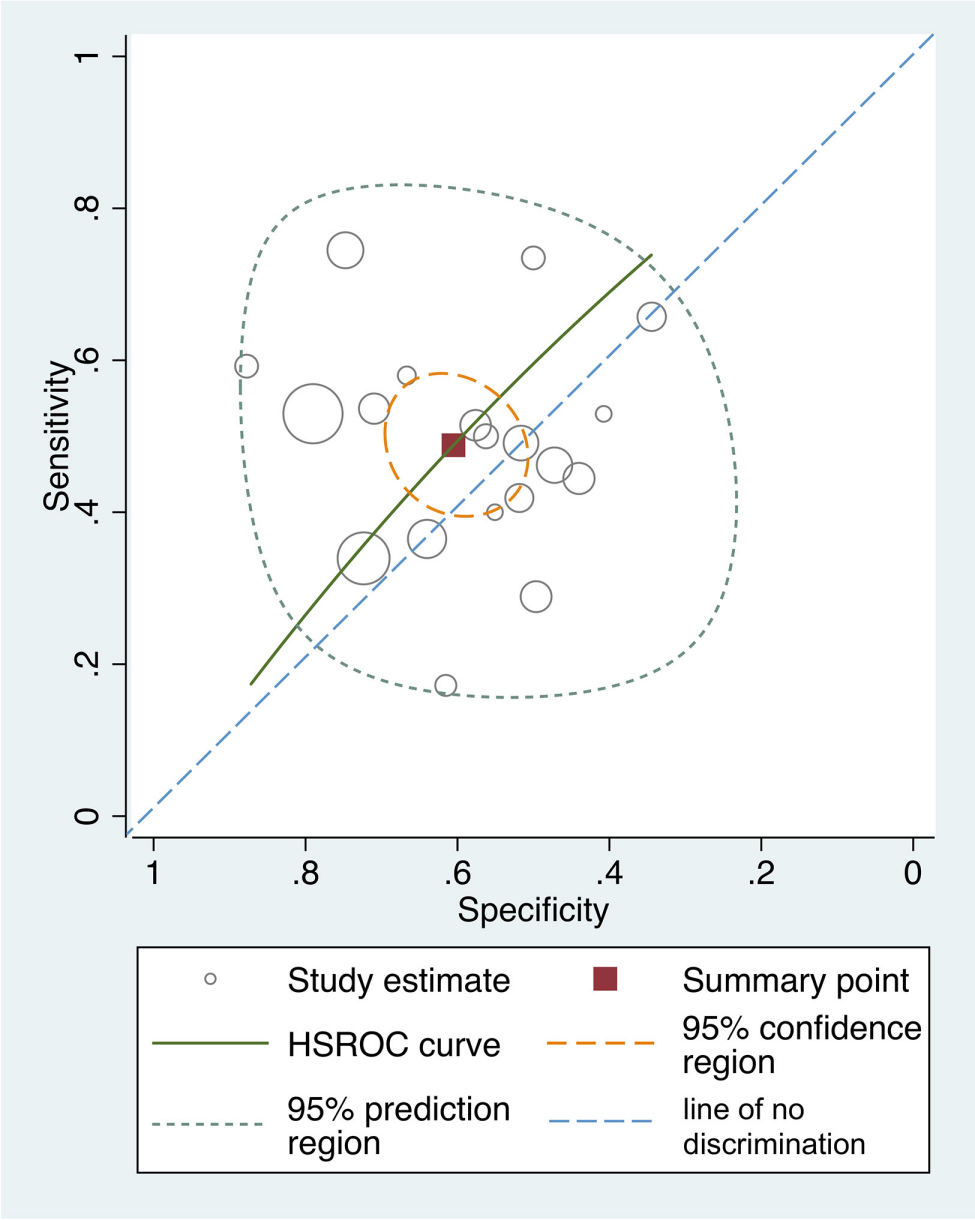
Hierarchical Summary Receiver Operating Characteristic Curve.

The pooled sensitivity was 0.49 (95% CI 0.42–0.56), the pooled specificity was 0.61 (95% CI 0.53–0.67), the pooled LR+ was 1.24 (95% CI 0.97–1.58), the pooled LR- was 0.85 (95% CI 0.70–1.02), and the DOR was 1.46 (0.95–2.26). In Fig 3, sensitivity and specificity of DRE shows great variation. The HSROC curve is close to the line of discrimination.

### Investigation of heterogeneity

Fig 2 shows that the heterogeneity among studies was large, with *I* ^*2*^ for sensitivity and specificity of 94% and 93%, respectively. The result of subgroup analyses is shown in Table 2. No significant differences were observed for the heterogeneity of sensitivity. On the other hand, whether the reference standard was only appendectomy or included follow-up with careful observation had a significant effect on heterogeneity in specificity. The subgroup analyses for age and sex of participants could not be performed because sufficient data for these analyses were not reported.

**Table 2:** The results of subgroup analyses.

	No. of studies	Sensitivity	*P*	Specificity	*P*
Publication year					
After 1990	10	0.47	0.84	0.63	0.66
Before 1990	9	0.51		0.57	
N					
> = 360	9	0.48	0.99	0.58	0.10
<360	10	0.50		0.64	
Study design					
prospective	9	0.52	0.48	0.67	0.98
retrospective	10	0.46		0.55	
Patient					
Children	2	0.56	0.78	0.81	0.37
Adult	4	0.42	0.78	0.62	0.74
Both	13	0.50		0.56	
Abdominal pain	3	0.41	0.67	0.72	0.96
Suspected appendicitis/Appendectomy	16	0.50		0.57	
Definition of DRE					
Right tenderness	8	0.51	0.68	0.57	0.13
Others	11	0.47		0.63	
Reference standard					
Appendectomy	10	0.47	0.88	0.52	<0.01
Appendectomy/follow-up	9	0.50		0.68	
Bias					
Without high risk of bias	9	0.45	0.62	0.56	0.05
At least one high risk of bias	10	0.52		0.65	

## Discussion

**Fig 4:**
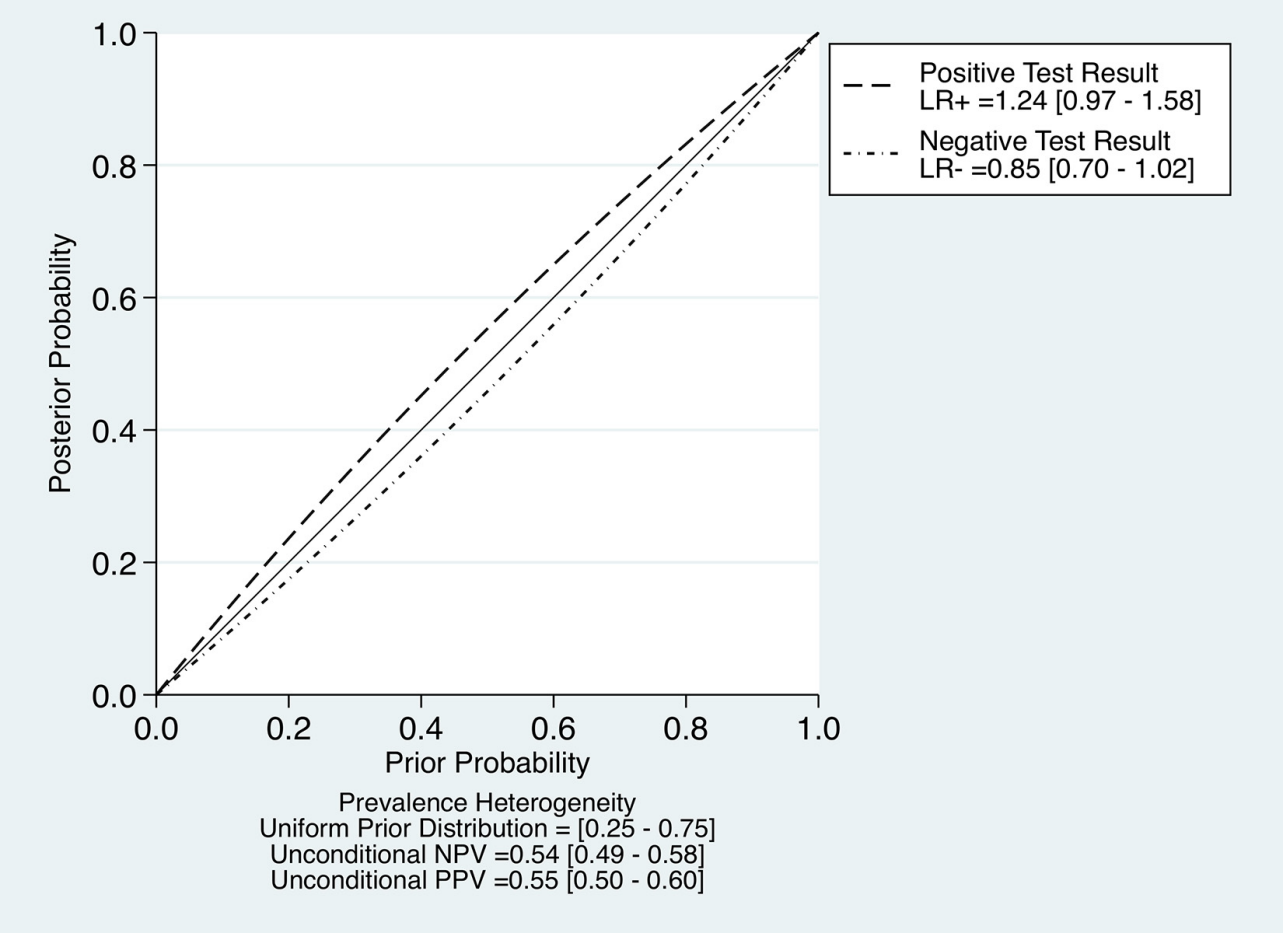
Relationship between pre and post-test probability based on the pooled LR+ and LR-.

Overall, our analysis showed poor diagnostic performance of DRE for acute appendicitis. Fig 4 shows the relationship between pre- and post-test probability based on the pooled LR+ and LR-. The change between pre- and post-test probability is minimal regardless of the results of DRE. These findings are consistent with the report that DRE results did not change physicians’ decisions regarding a diagnosis of appendicitis [46–48]. Considering the discomfort induced by DRE [10,11], the opinion that DRE is an indispensable, routine examination for evaluation of appendicitis should be reconsidered. On the other hand, the role of DRE should not be denied entirely. In *Schwartz’s Principles of Surgery*, it has been described as “When the appendix hangs into the pelvis, abdominal findings may be entirely absent, and the diagnosis may be missed. Right-sided rectal tenderness is said to help in this situation [49].” Thus, the DRE could have an important role when the inflamed appendix cannot be evaluated through abdominal examination. In our review, no study investigated such a specific situation. Instead, a report described the relationship between the sensitivity of DRE and the positions of inflamed appendixes [27]. The sensitivity for pelvic appendicitis was 0.38, while the sensitivity for all appendicitis was 0.44. The specificity was unknown. These data are not enough to conclude the role of DRE for pelvic appendicitis and further investigation is warranted.

Our analysis had several limitations. First, in an investigation of heterogeneity, the heterogeneity of sensitivity could not be explained by subgroup analyses. Many factors possibly contributing to heterogeneity could not be evaluated because they were not reported, such as the technique and experience of the physician and the position of the appendix. Second, we could not perform subgroup analyses defined a priori for age and sex because sufficient data for analyses were not obtained. Almost half of the retrieved studies were performed before the 1990s, and most authors contacted for further information did not have their data. It is known that the diagnosis of appendicitis is challenging in children younger than age 3 and elderly patients [50,51]. In women, there are a variety of other diagnoses, mostly related to gynecologic diseases, that mimic features of appendicitis. Therefore, the specificity of DRE could be lower. Thus, the diagnostic performance of DRE for these groups should be assessed separately. S1 Table shows some data for these groups, although there is not enough information to perform a meta-analysis. Third, it is preferable that all participants receive the same reference standard in diagnostic research. Partial verification bias may exist when a nonrandom set of patients does not undergo the reference standard. It usually leads to overestimation of sensitivity, and the effect on specificity varies [15]. However, it is ethically difficult to perform surgery on all participants, especially when the possibility of appendicitis is considered low. Therefore, we included studies that excluded appendicitis by clinical follow-up. In our review, 9 studies excluded appendicitis by appendectomy and clinical follow-up. Patients who do not undergo surgery and who do not develop appendicitis during follow-up are assumed not to have had appendicitis, but several studies suggest that spontaneous resolution is common [52,53]. Therefore, this might also lead to a high risk of bias. The sensitivity and specificity were 0.50 and 0.68 in the appendectomy/follow-up group, whereas they were 0.47 and 0.52, respectively, in the appendectomy group alone.

The strength of our systematic review is that this is the first systematic review and meta-analysis following a standard protocol and comprehensive search strategy on this topic. There have been several reviews without meta-analyses [8,9,12–15], and a review with a meta-analysis that did not follow a standard protocol [15]. The review with the meta-analysis investigated the value of various history, examinations, and laboratory tests, was not focused on DRE, and included only 5 studies [15].

In conclusion, acute appendicitis cannot be ruled in or out through the results of DRE. Reconsideration is warranted for the traditional teaching that DRE should be performed routinely in all patients with suspected appendicitis. Situations in which the DRE has an important role should be further investigated.

## Supporting Information

S1 Fig Summary of Risk of bias and applicability concerns. Review authors' judgements about each domain for each included study. The figure was generated using Review Manager Version 5.3.
(TIF)

S2 Fig Risk of bias and applicability concerns graph for included studies. Review authors' judgements about each domain presented as percentages across included studies. The figure was generated using Review Manager Version 5.3.
(TIF)

S3 Fig Funnel plot for publication bias assessment. (TIF)

S1 Table Diagnostic performance of DRE for woman and elderly in diagnosis of appendicitis. (DOCX)

S1 Text Study protocol and searching strategy. (DOCX)

S2 Text PRISMA checklist. (DOC)
