# Supplementary figures and images for: The Role of Digital Rectal Examination for Diagnosis of Acute Appendicitis: A Systematic Review and Meta-Analysis
Source: PLoS One. 2015 Sep 2;10(9):e0136996. doi: 10.1371/journal.pone.0136996 (PMC4557952; doi:10.1371/journal.pone.0136996)

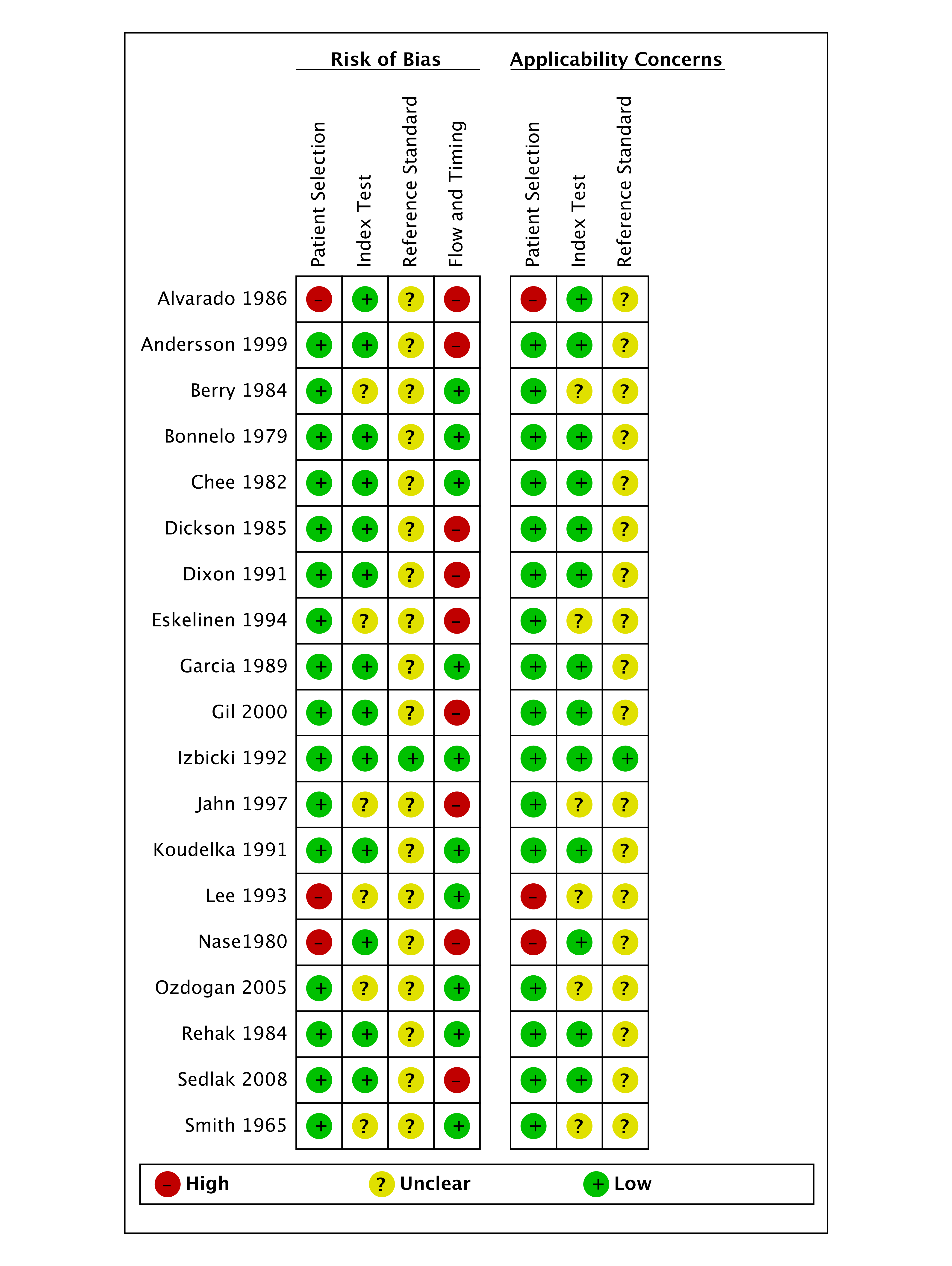

Supplement: S1 Fig — Review authors' judgements about each domain for each included study. The figure was generated using Review Manager Version 5.3. (TIF) [file pone.0136996.s001.tif]

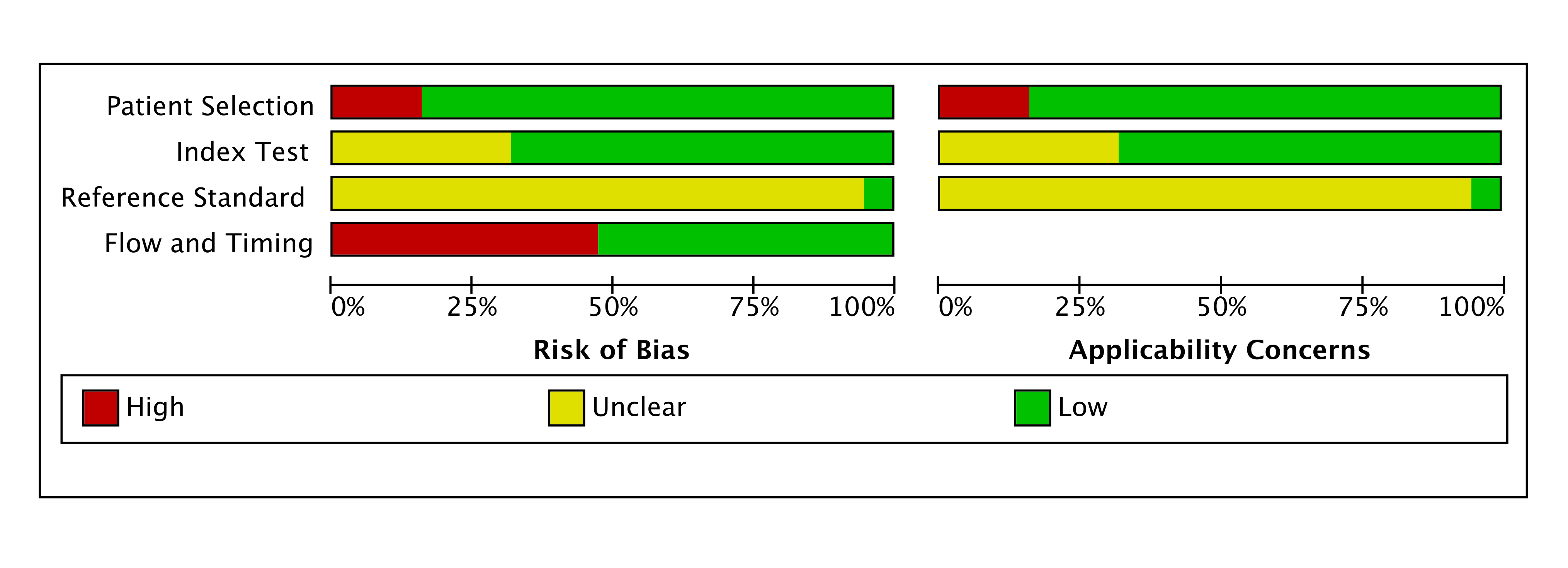

Supplement: S2 Fig — Review authors' judgements about each domain presented as percentages across included studies. The figure was generated using Review Manager Version 5.3. (TIF) [file pone.0136996.s002.tif]

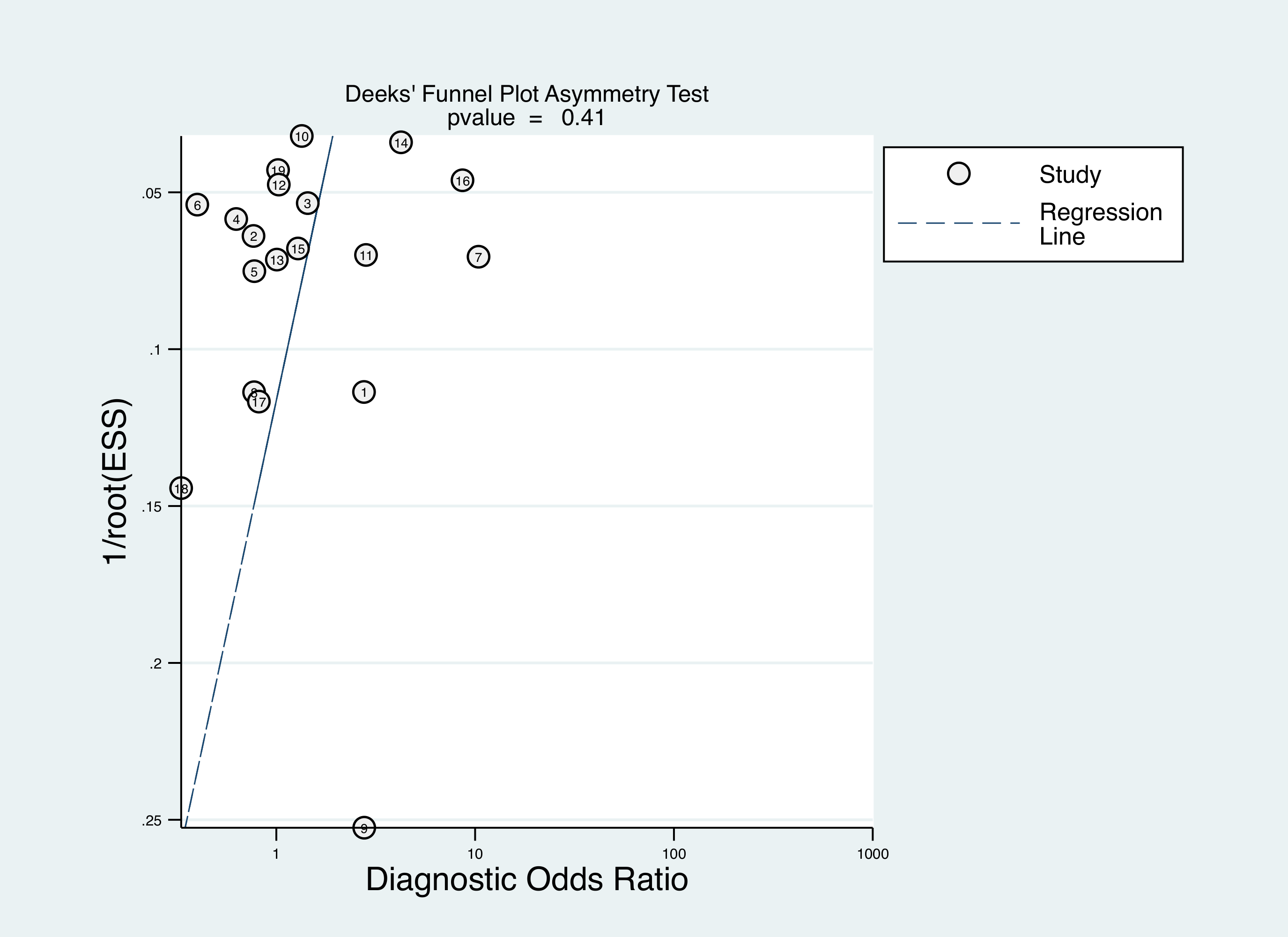

Supplement: S3 Fig — (TIF) [file pone.0136996.s003.tif]
