# Supplementary material for: The Role of Digital Rectal Examination for Diagnosis of Acute Appendicitis: A Systematic Review and Meta-Analysis
Source: PLoS One. 2015 Sep 2;10(9):e0136996. doi: 10.1371/journal.pone.0136996 (PMC4557952; doi:10.1371/journal.pone.0136996)
Supplement: S1 Table — (DOCX) [file pone.0136996.s004.docx]

|  | True positive | False positive | False negative | True negative | Sensitivity | Specificity | LR+ | LR- | DOR |
| --- | --- | --- | --- | --- | --- | --- | --- | --- | --- |
| Female |  |  |  |  |  |  |  |  |  |
| Bonello [25] | 88 | 27 | 100 | 22 | 0.47 | 0.45 | 0.85 | 1.18 | 0.72 |
| Ozdogen [44] | 18 | 51 | 5 | 3 | 0.78 | 0.06 | 0.83 | 3.91 | 0.21 |
|  |  |  |  |  |  |  |  |  |  |
| Elderly patients |  |  |  |  |  |  |  |  |  |
| Eskelinen [39] | 13 | 27 | 6 | 176 | 0.68 | 0.87 | 5.14 | 0.36 | 14.12 |
