# Supplementary material for: The Role of Digital Rectal Examination for Diagnosis of Acute Appendicitis: A Systematic Review and Meta-Analysis
Source: PLoS One. 2015 Sep 2;10(9):e0136996. doi: 10.1371/journal.pone.0136996 (PMC4557952; doi:10.1371/journal.pone.0136996)
Supplement: S1 Text — (DOCX) [file pone.0136996.s005.docx]

**Study protocol and searching strategies**

Study protocol for systematic review and meta-analysis to investigate the role of digital rectal examination (DRE) for diagnosis of acute appendicitis

**Objective**

To assess the role of DRE for diagnosis of acute appendicitis by the recommended methods of meta-analysis for diagnostic studies.

**Data Sources and Searches**

We will conduct a literature search of MEDLINE via PubMed, Scopus and Cochrane Library via Wiley Online Library from the earliest available date of indexing through November 23, 2014. The literature search will include potential studies in all languages. We will translate the non-English language papers and fully assess them for potential inclusion in the review as necessary. We will check reference lists of all included studies and relevant systematic review articles for additional references. We will contact authors of identified additional studies missed from the original electronic searches.

**Inclusion Criteria**

We will include the studies satisfying the following requirements.

**Types of studies**

(1) We will include studies assessing the digital rectal examination as an index test (the test under investigation) to evaluate the diagnosis of appendicitis.

(2) We will include studies that the reference standard (the criterion standard) of appendicitis was defined as histologically proven acute inflammation in the appendix.

(3) We will include studies that the exclusion of appendicitis were histologically proven or evaluated in the clinical follow-up with careful observation.

**Participants**

(1) We will include studies which included patients with abdominal pain or suspected appendicitis.

(2) We will include studies of patients with any age.

**Outcome measures**

It reported cases in absolute numbers of true-positive, false-positive, false-negative and true-negative results, or these data were derivable from the published results.

**Study selection**

Two authors (TT and NH) will examine the titles and abstracts of references identified by the electronic search strategies described below to check whether the study is likely to be relevant. Each potentially relevant study in the search will be obtained as a full article and independently assessed for inclusion by the same two review authors and, in the case of discordance, resolution will be sought by discussion between the two authors. The discordance in the selection of studies will be evaluated by quantifying both the percentage of agreement and Cohen’s Kappa (k). Values of kappa between 0.40 and 0.59 have been considered to reflect fair agreement, between 0.60 and 0.74 to reflect good agreement and 0.75 or more to reflect excellent agreement [1].

**Data extraction and Quality Assessment**

Two authors will use a structured, pilot-tested, Excel data collection form to independently extract the data from the included studies. Extracted data will include: study characteristics (Design, One/Two-gate, No of participants, No of excluded participants, Index test, Reference test, Blinding), patient characteristics (Setting, Age, Sex). The definition of children will be adopted in accordance with each article. Two authors will also independently assess methodological quality using the Quality Assessment of Diagnostic Accuracy Studies 2 (QUADAS-2) tool [2]. Any disagreement will be resolved by discussion between the two authors. When relevant information regarding design or outcomes is unclear, the study authors will be contacted for clarification.

**Data Synthesis and Analysis**

To evaluate the diagnostic performance, we will construct 2 × 2 tables. Measures of the diagnostic performance, including sensitivity, specificity, positive likelihood ratio (LR+), negative likelihood ratio (LR-) and diagnostic odds ratios (DORs), will be reported as point estimates with 95% confidence intervals (CI). A DOR can be calculated as the ratio of the odds of positivity in a disease state relative to the odds of positivity in the nondisease state, with higher values indicating better discriminatory test performance. The value of the DOR ranges from zero to infinity, with higher values indicative of better discriminative performance. A value of 1 indicates that the test does not discriminate between people with and without the disease/condition [3]. Publication bias will be assessed using the effective sample size funnel plot and associated regression test of asymmetry [4]. We will use the bivariate random-effects model for analysis and pooling of the diagnostic performance measures across studies [5]. The bivariate model will be used to estimate pairs of logit transformed sensitivity and specificity from studies, incorporating the correlation that might exist between sensitivity and specificity. We will use the hierarchical summary receiver operating characteristic curves (HSROC) to estimate the area under the curve [6]. Between-study statistical heterogeneity will be assessed using *I2* test on the basis of the random-effects analysis [7]. To explain heterogeneity, we will perform meta-regression to identify potential sources of bias. Pooled estimates will also be calculated for subgroups of studies that were defined according to specific study designs. The following variables are selected a priori as potential sources of heterogeneity: publication year, no. of participants, study design (prospective vs. retrospective), age of participants (children vs. adults vs. all ages), sex, inclusion criteria (abdominal pain vs. suspected appendicitis/appendectomy), definition of positive DRE (right-sided tenderness vs. the others), reference standard (appendectomy vs. appendectomy/follow-up). Two-sided P < .05 will be considered statistically significant. Statistical analyses will be performed with commercial software programs (STATA, version SE ; StataCorp LP).

**Search Strategies**

**CENTRAL via Wiley Online Library**

#1 MeSH descriptor: [acute abdomen] explode all trees

#2 “acute abdomen”

#3 MeSH descriptor: [appendicitis] explode all trees

#4 “appendicitis”

#5 MeSH descriptor: [abdominal pain] explode all trees

#6 “abdominal pain”

#7 #1 or #2 or #3 or #4 or #5 or #6

#8 MeSH descriptor: [Digital rectal examination] explode all tree

#9 "rectal examination”

#10 #8 OR #9

#11 #7 AND #10

**MEDLINE via PubMed**

#1 “Digital rectal examination[mh]”

#2 “rectal examination”

#3 #1 OR #2

#4 “Abdomen, Acute[mh]”

#5 “acute abdomen”

#6 “appendicitis[mh]”

#7 “appendicitis”

#8 “abdominal pain[mh]”

#9 “abdominal pain”

#10 #4 OR #5 OR #6 OR #7 OR #8 OR #9

#11 #3 AND #10

**SCOPUS**

#1 TITLE-ABS-KEY(“rectal examination”)

#2 TITLE-ABS-KEY(“acute abdomen” OR “appendicitis” OR “abdominal pain”)

#3 #1 AND #2

**References**

1. Bland JB, Altman DG. Statistical methods for assessing agreement between two methods of clinical measurement. Lancet. 1986;1: 307-310.

2. Whiting P, Rutjes AW, Reitsma JB, Bossuyt PM, Kleijnen J. The development of QUADAS: a tool for the quality assessment of studies of diagnostic accuracy included insystematic reviews. BMC Med Res Methodol. 2003;3: 25.

3. Glas AS, Lijmer JG, Prins MH, Bonsel GJ, Bossuyt PM. The diagnostic odds ratio: a single indicator of test performance. J Clin Epidemiol. 2003;56: 1129-1135.

4. Deeks JJ, Macaskill P, Irwig L. The performance of tests of publication bias and other sample size effects in systematic reviews of diagnostic test accuracy was assessed. J Clin Epidemiol. 2005;58: 882-893.

5. Reitsma JB, Glas AS, Rutjes AW, Scholten RJ, Bossuyt PM, Zwinderman AH. Bivariate analysis of sensitivity and specificity produces informative summary measures in diagnostic reviews. J Clin Epidemiol. 2005;58: 982-990.

6. Rutter CM, Gatsonis CA. A hierarchical regression approach to metaanalysis of diagnostic test accuracy evaluations. Stat Med. 2001;20: 2865-2884.

7. Thompson SG. Why sources of heterogeneity in meta-analysis should be investigated. BMJ. 1994;309: 1351-1355.
